# Supplementary material for: A mechanistic reinterpretation of fast inactivation in voltage-gated Na+ channels
Source: Nat Commun. 2023 Aug 21;14:5072. doi: 10.1038/s41467-023-40514-4 (PMC10442390; doi:10.1038/s41467-023-40514-4)
Supplement: Supplementary file 1 — Supplementary Information [file 41467_2023_40514_MOESM1_ESM.pdf]

Supplemental information titles and legends

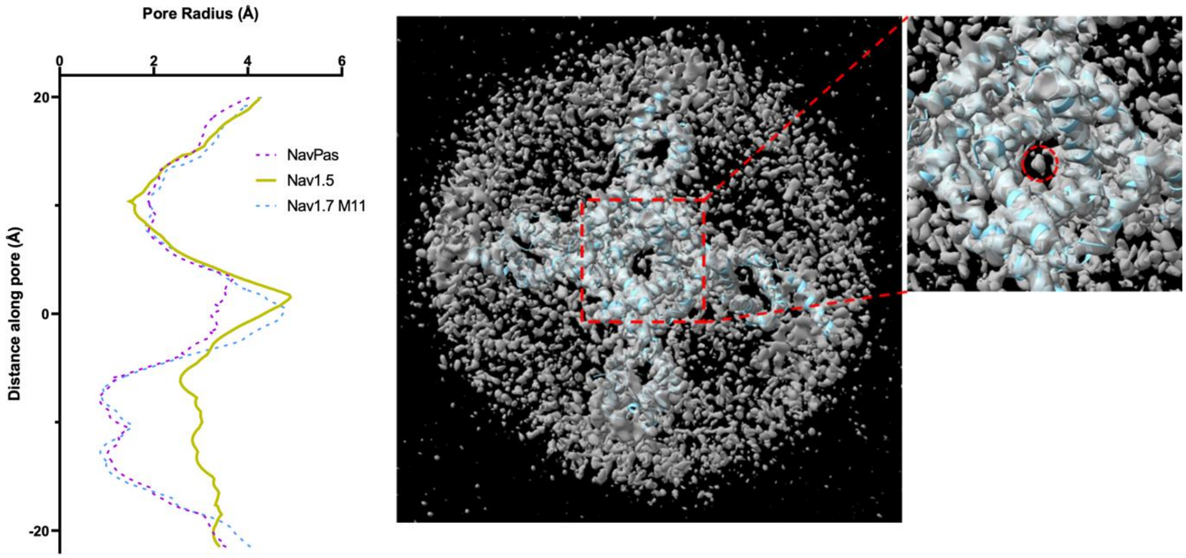

Supplementary Figure 1. Differences in pore radius between NavPas, Nav1.7M11 and Nav1.5 (6UZ3). A) The narrowest part in NavPas and Nav1.7 M11 structure is much broader than the Nav1.5 structure. B) Intracellular view of the electron density map overlayed with the structure model. There exists unidentified density inserting into the pore (highlighted in red circle).

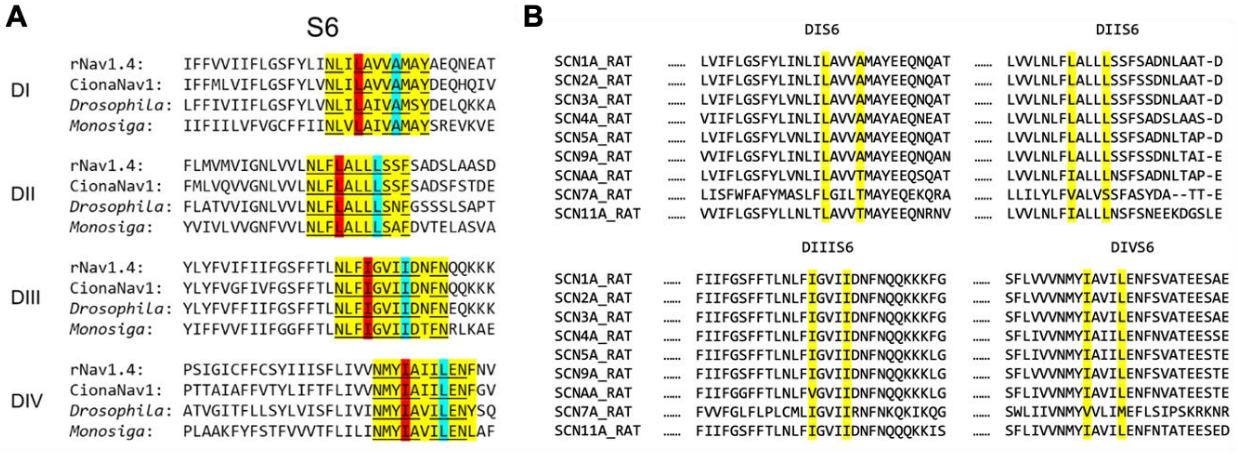

Supplementary Figure 2. Sequence alignment of Nav from different species (1). (A) and different rat isoforms (B). The identified residues are very conserved across many different species.

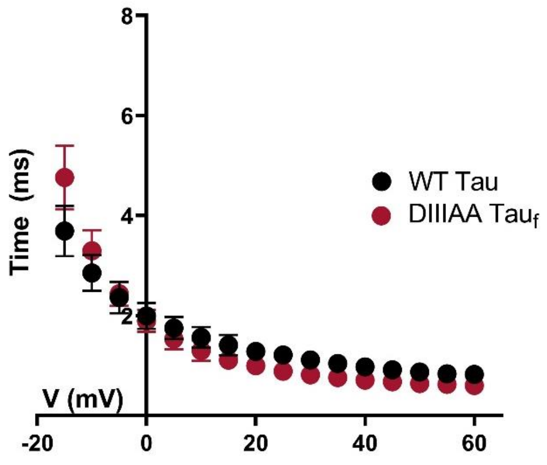

Supplementary Figure 3. Fast Inactivation time constant for WT (black, N = 5) and DIIIAA (red, N = 4). Fast inactivation kinetics were characterized by fitting a single exponential function for WT and two-exponential function for DIIIAA. Data are shown as Mean ± SEM.

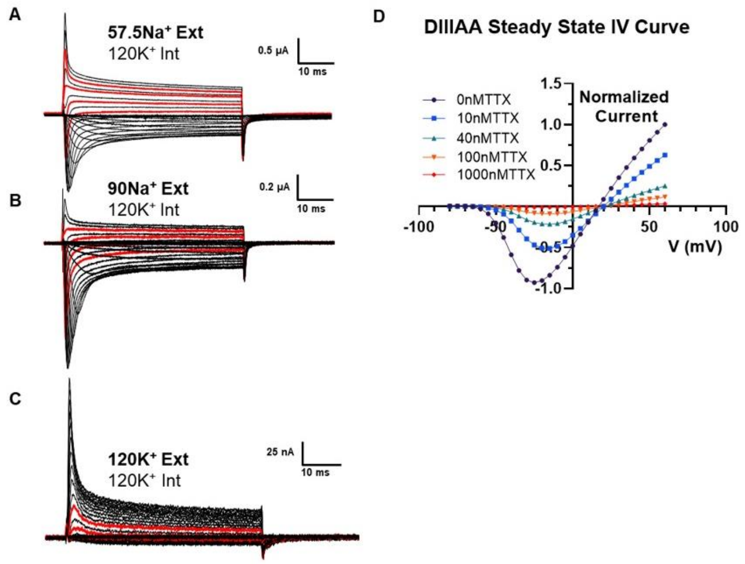

Supplementary Figure 4. DIIIAA ionic currents elicited by a set of voltage pulses (from -80 to + 60 mV every 5 mV), using 57.5 mM external Na (A), 90 mM external Na (B) or 120 external K (C). The red lines indicate the traces used. TTX could block the current efficiently (D).

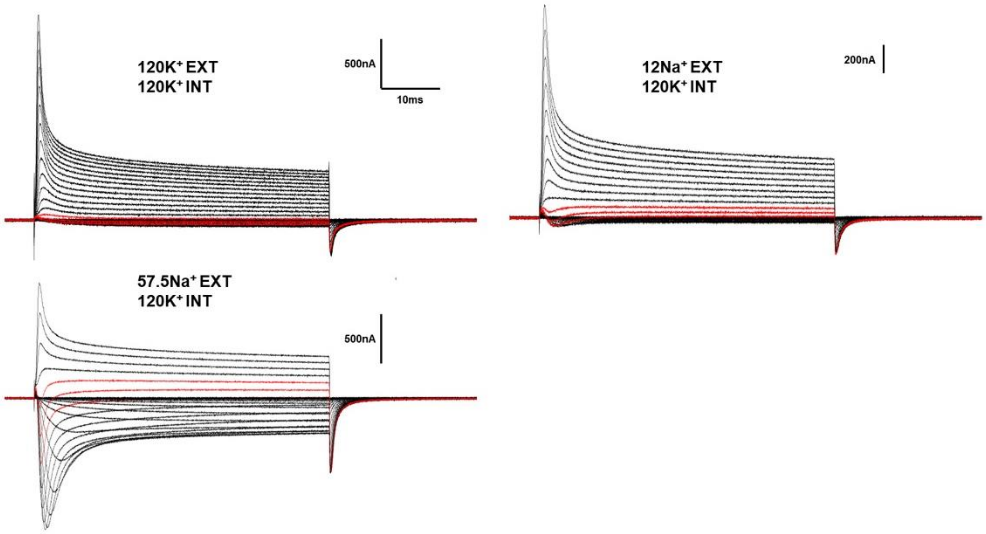

Supplementary Figure 5. Once K^+ was exchanged by Na^+ in the external solution, the crossing reappears, showing the reversibility of the two components of current. The experiment shown above was performed on the same oocyte starting with external $120K^+$ then to $12Na^+$ and finally in $57.5Na^+$. The traces near the reversible potential are highlighted in red.

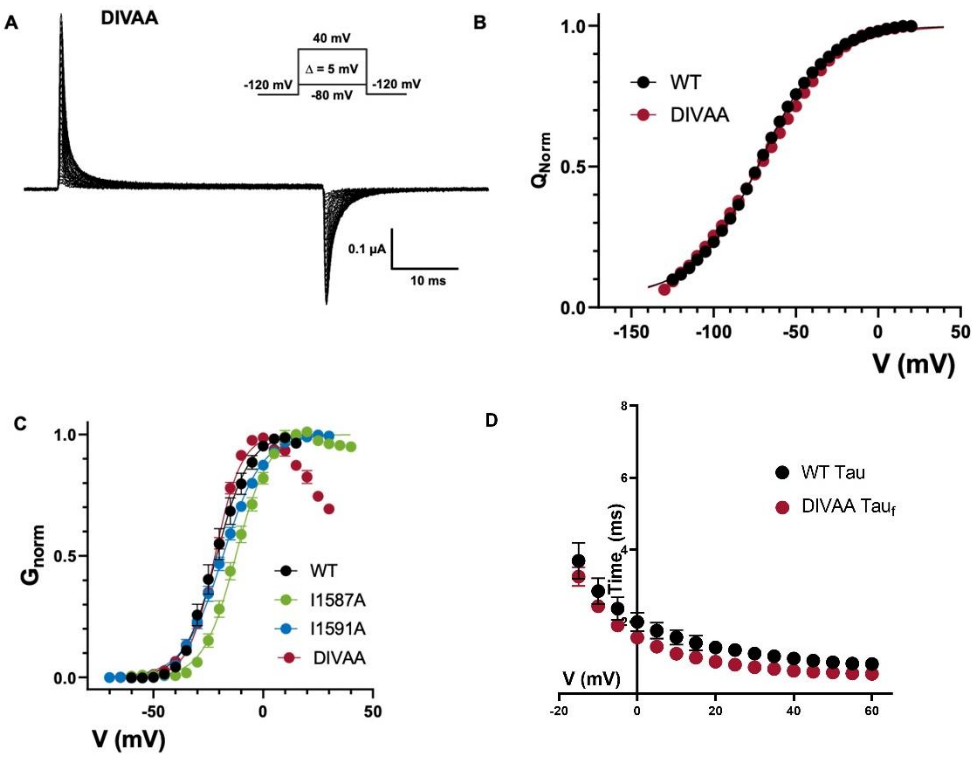

Supplementary Figure 6: Properties of double alanine mutations at DIV S6. A) Representative gating current traces for DIVAA. Inset is the voltage protocol. B) Q-V curves for WT (black, N = 6) and DIVAA (red, N = 7). C) G-V curves for WT (black, N = 4), I1587A (green, N = 5), L1591A (blue, N = 5) and DIVAA (red, N = 6). G-V curves were calculated from the peak for WT, I1587A and L1591A whereas for DIVAA it was calculated from the steady state currents. D) Fast Inactivation time constant for WT (black, N = 5) and DIVAA (red, N = 5). Fast inactivation kinetics were characterized by fitting a single exponential function for WT and two-exponential function for DIIIAA. Data are shown as Mean ± SEM.

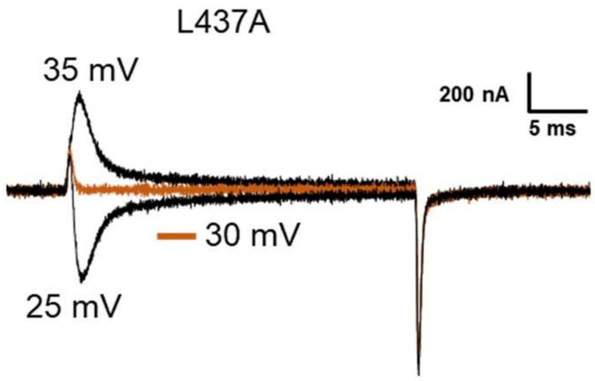

Supplementary Figure 7: Lack of two components on DIA ionic currents. Representative ionic current traces for DIA at 3 voltages close to the reversal potential (~30 mV).

Supplementary Table 1: Fit parameters of the voltage-dependence of inactivation for all the channels tested.

	$V_{I\_1/2}$	z_I	Base	n
WT	-48.83±0.18	-3.08	/	8
I1284A	-37.10±0.32	-2.96	0.01±0.0069	6
I1288A	-38.82±0.27	-3.37	0.01±0.0060	6
DIIIAA	-41.52±0.26	-3.51	0.13±0.0064	4
I1587A	-47.58±0.13	-3.22	0.00±0.0026	5
I1591A	-40.34±0.20	-4.04	0.01±0.0046	5
DIVAA	-51.62±0.32	-3.02	0.07±0.0069	8

Supplementary Table 2: Fit parameters of the voltage-dependence of activation for all the channels tested.

	$V_{G\_1/2}$	z_G	n
WT	-21.19±0.45	3.30	4
I1284A	-5.951±0.33	3.42	4
I1288A	-11.62±0.32	2.97	6
DIIIAA	-29.72±0.62	2.67	4
IQM	-23.89±0.26	4.97	4
IQM_DIIIAA	-11.65±0.38	4.68	4
I1587A	-12.48±0.31	3.33	5
I1591A	-18.31±0.27	2.75	5

**DIVAA**	**-21.97±0.60**	**4.05**	**6**
**L437A**	**-7.511±0.30**	**2.64**	**8**

Supplementary Table 3: Fit parameters of the voltage-dependence of charge movement.

	$V_{Q\_1/2}$	z_Q	**n**
**WT**	**-74.12±0.28**	**1.16**	**6**
**DIIIAA**	**-66.34±0.61**	**1.12**	**6**
**DIVAA**	**-73.13±0.23**	**1.07**	**7**

Supplementary Reference

1. Liebeskind, B.J., D.M. Hillis, and H.H. Zakon. 2011. Evolution of sodium channels predates the origin of nervous systems in animals. *Proc. Natl. Acad. Sci. U. S. A.* 108:9154–9159.
