# Supplementary material for: A mechanistic reinterpretation of fast inactivation in voltage-gated Na+ channels
Source: Nat Commun. 2023 Aug 21;14:5072. doi: 10.1038/s41467-023-40514-4 (PMC10442390; doi:10.1038/s41467-023-40514-4)
Supplement: Supplementary file 3 — Reporting Summary [file 41467_2023_40514_MOESM3_ESM.pdf]

nature portfolio

Corresponding author(s): Francisco Bezanilla

Last updated by author(s): Jun 30, 2023

Reporting Summary

Nature Portfolio wishes to improve the reproducibility of the work that we publish. This form provides structure for consistency and transparency in reporting. For further information on Nature Portfolio policies, see our Editorial Policies and the Editorial Policy Checklist.

Statistics

For all statistical analyses, confirm that the following items are present in the figure legend, table legend, main text, or Methods section.

n/a	Confirmed	
☐	☒	The exact sample size (*n*) for each experimental group/condition, given as a discrete number and unit of measurement
☒	☐	A statement on whether measurements were taken from distinct samples or whether the same sample was measured repeatedly
☐	☒	The statistical test(s) used AND whether they are one- or two-sided<br>*Only common tests should be described solely by name; describe more complex techniques in the Methods section.*
☒	☐	A description of all covariates tested
☒	☐	A description of any assumptions or corrections, such as tests of normality and adjustment for multiple comparisons
☐	☒	A full description of the statistical parameters including central tendency (e.g. means) or other basic estimates (e.g. regression coefficient) AND variation (e.g. standard deviation) or associated estimates of uncertainty (e.g. confidence intervals)
☒	☐	For null hypothesis testing, the test statistic (e.g. *F*, *t*, *r*) with confidence intervals, effect sizes, degrees of freedom and *P* value noted<br>*Give P values as exact values whenever suitable.*
☒	☐	For Bayesian analysis, information on the choice of priors and Markov chain Monte Carlo settings
☒	☐	For hierarchical and complex designs, identification of the appropriate level for tests and full reporting of outcomes
☒	☐	Estimates of effect sizes (e.g. Cohen's *d*, Pearson's *r*), indicating how they were calculated

Our web collection on statistics for biologists contains articles on many of the points above.

Software and code

Policy information about availability of computer code

Data collection	We used a in-house developed acquisition software, GPatchMC64.exe (Ver. 1.1)
Data analysis	We used a in-house developed analysis software, Analysis.exe (Ver. 2.0), to measure, fit and integrate the current traces, Excel (version 2208), Graphpad Prism 9, MATLAB (R2020b) to plot and fit the curves, ChimeraX (1.2.5), VMD to analyze the structural data, Snapgene (Version 7.0) to align the sequences.

For manuscripts utilizing custom algorithms or software that are central to the research but not yet described in published literature, software must be made available to editors and reviewers. We strongly encourage code deposition in a community repository (e.g. GitHub). See the Nature Portfolio guidelines for submitting code & software for further information.

Data

Policy information about availability of data

All manuscripts must include a data availability statement. This statement should provide the following information, where applicable:

- Accession codes, unique identifiers, or web links for publicly available datasets
- A description of any restrictions on data availability
- For clinical datasets or third party data, please ensure that the statement adheres to our policy

All data generated and resources used in this study will be made available to readers upon request. The reason that we did not choose to deposit all the raw data acquired directly is that the electrophysiological data is in our own format that would require a long processing to make it into standard format, such as ascii. On the

other hand, if somebody request some of the data we can perform the format change for the specific data of interest.
The protein structures analyzed in this studies can be accessed in protein data bank. NavPas PDB: 6A95; Nav1.7 M11 PDB: 7XVF; Nav1.5 PDB: 6UZ3

Research involving human participants, their data, or biological material

Policy information about studies with human participants or human data. See also policy information about sex, gender (identity/presentation), and sexual orientation and race, ethnicity and racism.

Reporting on sex and gender	NA
Reporting on race, ethnicity, or other socially relevant groupings	NA
Population characteristics	NA
Recruitment	NA
Ethics oversight	NA

Note that full information on the approval of the study protocol must also be provided in the manuscript.

Field-specific reporting

Please select the one below that is the best fit for your research. If you are not sure, read the appropriate sections before making your selection.

☒ Life sciences ☐ Behavioural & social sciences ☐ Ecological, evolutionary & environmental sciences

For a reference copy of the document with all sections, see nature.com/documents/nr-reporting-summary-flat.pdf

Life sciences study design

All studies must disclose on these points even when the disclosure is negative.

Sample size	No sample size calculation was performed. We follow the standards in the field to include at least 4 independent measurements of any given mutant and experimental protocol.
Data exclusions	Oocytes with high leak current or low expression were excluded from analysis.
Replication	Oocytes from different batches were used to replicate the results and in all cases the results are well replicated. All the mutants and experimental protocols were repeated in at least 4 different oocytes from at least 2 different batches.
Randomization	All the oocytes were selected randomly for mRNA injection and the injected oocytes in good health were selected randomly for recordings.
Blinding	We did not utilize double blind experimental design because the phenotypically, the differences among the mutants and the wild type are big. And the analysis was done systematically.

Reporting for specific materials, systems and methods

We require information from authors about some types of materials, experimental systems and methods used in many studies. Here, indicate whether each material, system or method listed is relevant to your study. If you are not sure if a list item applies to your research, read the appropriate section before selecting a response.

Materials & experimental systems

n/a	Involved in the study	
☒	☐	Antibodies
☒	☐	Eukaryotic cell lines
☒	☐	Palaeontology and archaeology
☒	☐	Animals and other organisms
☒	☐	Clinical data
☒	☐	Dual use research of concern
☒	☐	Plants

Methods

n/a	Involved in the study	
☒	☐	ChIP-seq
☒	☐	Flow cytometry
☒	☐	MRI-based neuroimaging
